# PTBP1 plays an important role in the development of gastric cancer

**DOI:** 10.1186/s12935-023-03043-0

**Published:** 2023-09-05

**Authors:** Zewen Chu, Miao Zhu, Yuanyuan Luo, Yaqi Hu, Xinyi Feng, Haibo Wang, Masataka Sunagawa, Yanqing Liu

**Affiliations:** 1 The Affiliated Hospital of Yangzhou University, Yangzhou University, Yangzhou, China; 2 Institute of Translational Medicine, Medical College, Yangzhou University, Yangzhou, China; 3 The Key Laboratory of Syndrome Differentiation and Treatment of Gastric Cancer of the State Administration of Traditional Chinese Medicine, Yangzhou, China; 4 Department of physiology, School of Medicine, Showa University, Tokyo, Japan

**Keywords:** PTBP1:Polypyrimidine Tract binding protein 1, Gastric cancer, Proliferation, Actin cytoskeleton remodeling, PTBP1 Cas9-KO mouse model

## Abstract

**Background:**

Polypyrimidine tract binding protein 1 (PTBP1) has been found to play an important role in the occurrence and development of various tumors. At present, the role of PTBP1 in gastric cancer (GC) is still unknown and worthy of further investigation.

**Methods:**

We used bioinformatics to analyze the expression of PTBP1 in patients with GC. Cell proliferation related experiments were used to detect cell proliferation after PTBP1 knockdown. Skeleton staining, scanning electron microscopy and transmission electron microscopy were used to observe the changes of actin skeleton. Proliferation and actin skeleton remodeling signaling pathways were detected by Western Blots. The relationship between PTBP1 and proliferation of gastric cancer cells was further detected by subcutaneous tumor transplantation. Finally, tissue microarray data from clinical samples were used to further explore the expression of PTBP1 in patients with gastric cancer and its correlation with prognosis.

**Results:**

Through bioinformatics studies, we found that PTBP1 was highly expressed in GC patients and correlated with poor prognosis. Cell proliferation and cycle analysis showed that PTBP1 down-regulation could significantly inhibit cell proliferation. The results of cell proliferation detection related experiments showed that PTBP1 down-regulation could inhibit the division and proliferation of GC cells. Furthermore, changes in the morphology of the actin skeleton of cells showed that PTBP1 down-regulation inhibited actin skeletal remodeling in GC cells. Western Blots showed that PTBP1 could regulate proliferation and actin skeleton remodeling signaling pathways. In addition, we constructed PTBP1 Cas9-KO mouse model and performed xenograft assays to further confirm that down-regulation of PTBP1 could inhibit the proliferation of GC cells. Finally, tissue microarray was used to further verify the close correlation between PTBP1 and poor prognosis in patients with GC.

**Conclusions:**

Our study demonstrates for the first time that PTBP1 may affect the proliferation of GC cells by regulating actin skeleton remodeling. In addition, PTBP1 is closely related to actin skeleton remodeling and proliferation signaling pathways. We suppose that PTBP1 might be a potential target for the treatment of GC.

**Graphical abstract:**

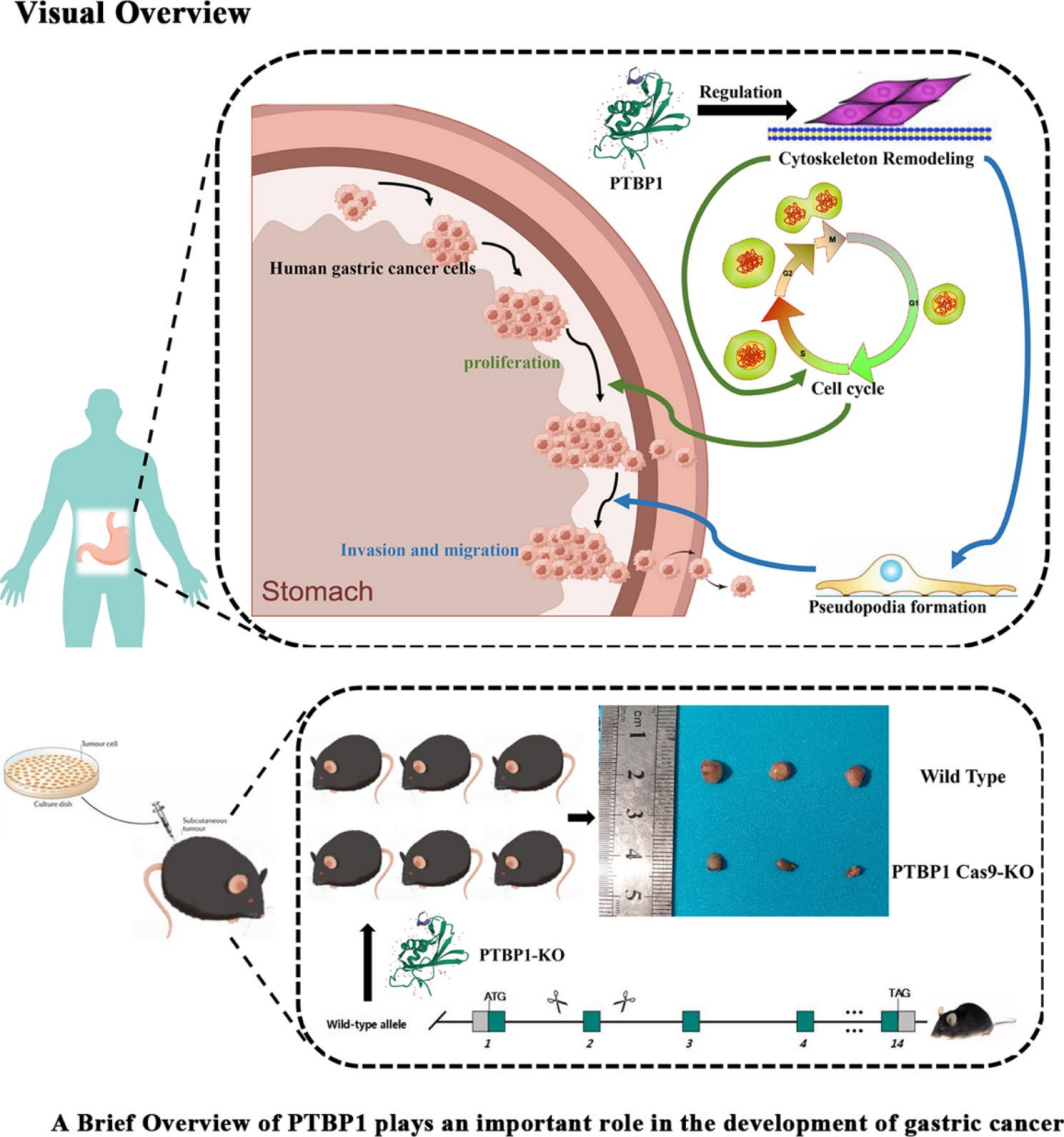

**Supplementary Information:**

The online version contains supplementary material available at 10.1186/s12935-023-03043-0.

## Background

Gastric cancer (GC) is a common gastrointestinal malignancy, which ranks the fifth among all malignancies in terms of incidence, and the fourth among cancer-related deaths in terms of mortality [1]. The clinical manifestations of GC are not significantly specific. Similar to the manifestations of non-malignant digestive tract diseases, the onset of GC is insidious, the progression is rapid, early diagnosis is difficult, and the diagnosis is mostly in the middle and late stage, which makes most cases lose the best opportunity for surgery [2]. The clinical manifestations of middle and advanced GC are mostly accompanied by adjacent tissue or organ invasion, intraperitoneal lymph node metastasis and organ metastasis, and the prognosis is poor. Early diagnosis is very important to improve the prognosis of patients. Therefore, it is necessary to discover molecules associated with GC for its treatment [3].

Actin skeleton remodeling is closely related to proliferation, invasion and metastasis of GC cells [4–6]. Therefore, it is of great significance to study how to inhibit actin cytoskeleton remodeling in GC cells to solve the clinical problems of GC recurrence and metastasis. PTBP1 preferentially binds to polypyrimidine-rich stretches of RNA. It functions mainly in splicing, and can shuttle between the cytoplasm and the nucleus [7]. PTBP1 is a common RNA-binding protein that plays an important role in the occurrence and development of a variety of diseases. Studies have shown that PTBP1 in breast cancer and lung cancer cells can increase the metastatic power and invasion ability of breast cancer and lung cancer cells by affecting the assembly of F-actin and the formation of pseudopodia, and promote the occurrence of metastatic tumors [8, 9]. However, whether PTBP1 can promote the proliferation, invasion and metastasis of GC cells by regulating actin skeleton remodeling has not been determined, which is worthy of further verification and exploration.

## Methods

### Cell lines

AGS and MKN-28 cells were purchased from the Procell Life Science&Technology Co.,Ltd. (Wuhan, China, Cat. no. CL-0022, Cat. no. CL-0291). All cells were identified by STR, and STR typing showed no cross contamination of human cells in the cell lines. No mycoplasma was detected in the two cells. Cells were cultured in RPMI 1640 (HyClone, USA, Cat. no. SH30809.01) containing 10% fetal bovine serum (FBS) (TransGen Biotech, China, Cat. no. FS301) and the culture dishes (Corning, USA, Cat. no. 150,464) were placed in an incubator containing 5% CO_2_ at a constant temperature of 37℃. After the cells were overgrown in the culture dishes, the cells were digested and subcultured with 0.25% trypsin digestion solution (Beyotime Biotechnology, China, Cat. no. C0201).

### Construction of lentiviral vector and lentiviral transfection

GC cells were seeded uniformly into 24-well plates (Corning, USA, Cat. no. 3524) and placed in an incubator overnight. Then, they were transfected with lentiviral vectors encoding small interfering RNA targeting PTBP1 (LV-PTBP1-RNAi) and control lentivirus CON207 (U6-MCS-Ubiquitin-Cherry-ires-Puromycin). PTBP1 (LV-PTBP1-Rnai) and control lentivirus CON207 (U6-MCS-Ubiquitin-Cherry-ires-Puromycin) were synthesized by Shanghai Genechem Co., Ltd. (Shanghai, China). Transfection reagent HitransG (A&P Set) were provided by Shanghai Genechem Co., Ltd. (Shanghai, China). The results of virus transfection were detected by fluorescence inverted microscopy (Olympus, Japan), and Western blot.

### CCK-8 assay

Use the Cell Counting Kit 8 (Abcam, UK, Cat. no. ab228554). Plate 3000 cells per well in 96-well plates (Corning, USA, Cat. no. 3599). Add test compounds into cells and incubate for a desired period (24, 48, 72, 96 and 120 h). The volume is 100 µl for a 96 well plate. Add 10 µl/well of CCK8 Solution to each well. Protect from the light and incubate for 1–4 h at 37ºC. The absorbance at 460 nm was measured with an enzyme marker (PerkinElmer, USA).

### EdU cell proliferation assay

Experiments were performed according to the BeyoClick™ EdU Cell Proliferation Assay Kit (Beyotime Biotechnology, China, Cat. no. C0078S) instructions. After Edu labeling of cells, the culture medium was removed, and 1ml 4% paraformaldehyde was added to fix the cells, and the cells were fixed for 15 min. The fixing solution was removed, and after washing, 1ml 0.1% Triton X-100 was added to each well and incubated for 10–15 min at room temperature. After washing, the nuclei were stained with DAPI. The results were observed and recorded with a fluorescent inverted microscope. Images were acquired under a microscope at 10x magnification.

### Cell cycle analysis

After removing the culture medium, the cells were digested by adding trypsin digestion solution. The suspension was transferred to a centrifuge tube, centrifuged at 1000 g for 5 min, and the supernatant was discarded to retain the cell precipitation. Then the cell precipitation was washed 1–2 times with pre-cooled PBS buffer, and the supernatant was discarded by centrifugation in the same way to retain the cell precipitation. Add 1 mL of precooled 75% ethanol to the collected cell precipitate sample, and gently blow the cells to make full contact. The cells were fixed at 4 ° C for 30 min. Add 500 µL of the prepared staining working solution (Servicebio, China, Cat. no. G1700), gently blow to disperse the cells and mix with the staining working solution. The cells were incubated at 37℃ for 30 min in the dark, and the red fluorescence was detected by flow cytometry at the excitation wavelength of 488 nm.

### Colony formation assay

Cells were counted and then planted in 60 mm dish (Corning, USA, Cat. no. 150,462), distributed evenly, cultured continuously for 2 weeks. In order to prevent the medium from drying and evaporating in the long-term culture process, the fresh medium was replaced during the culture period. After cleaning with PBS, the cells were fixed with paraformaldehyde and stained with crystal violet solution. The results were counted with ImageJ software (National Institutes of Health, USA).

### Wound healing assay

The linear scratch wounds were created on the confluent cell monolayers using a pipette tip and the exfoliated cells were removed with phosphate-buered saline (PBS) (Beyotime Biotechnology, China, Cat. no. C0221A). Take photos according to the time designed by the experiment. The degree of wound healing (%), calculated as [(scratch width of the shNC group-scratch width of the shPTBP1 group)/scratch width of the shNC group] x 100%, was used to measure the migration capacity of cells. Images were acquired under a microscope at 20x magnification.

### Cell proliferation and movement were analyzed by high-content cell analyzer

The number of two GC cells was adjusted to 4 × 10^3^ cells per well, and the cells were seeded in 96-well plates. After incubation at 37 ℃ for 12 h, the samples were transferred to PerkinElmer Operetta CLS High-Content Imaging System (PerkinElmer, USA). The observation was continued for 12 h. The system will automatically track cell division and proliferation. Harmony 4.1 software was used for data collection and analysis.

### Cytoskeleton staining

According to the instructions of the tetramethylrhodamine (TRITC)‑conjugated Phalloidin (Merck, Germany, Cat. no. FAK100), drop the cell suspension on the sterilized glass slide, and fix the slide in 4% formaldehyde after climbing the slide in the incubator for 24 h. 0.1% Triton X-100 was permeabilized, TRITC-labeled phalloidin was added dropwise. After incubation with DAPI staining solution, the results were observed by laser confocal microscopy (Nikon, Japan). Images were acquired under a microscope at 1000x magnification.

### Scanning electron microscopy (SEM)

The cultured cells will be spread, discard the culture medium, and then the crawling slices will be placed in a 6-well plate, the plates were washed twice with PBS, fixed with 1.5 mL of 2.5% glutaraldehyde fixing solution added to each well, and then rinsed with PBS 3 times, fixed with 1% OsO_4_, and dehydrated ∶ (acetone ∶ isoamyl acetate) (1:1). Isoamyl acetate, critical point drying, ion sputtering instrument sprayed gold, scanning electron microscope (HITACHI, Japan) at 1.00 K, 3.00Kx magnification, photography.

### Transmission electron microscopy (TEM)

After cell treatment, the cells were collected and fixed with 2.5% glutaraldehyde at 4 ℃. The cells were gently washed with pre-cooled PBS. Cells were completely immersed by adding 1% osmic acid and fixed for 2 h. Then, 50% ethanol was added and soaked, and then 70% ethanol was further soaked and placed at 4℃ overnight. At room temperature, different concentrations of ethanol (80%, 90%, 95%, 100%) were used for gradient dehydration, and each step was 15 min. After absorption of ethanol, add 100% acetone and leave at room temperature. After centrifugation, discard the supernatant, add 100% acetone (containing anhydrous Na_2_SO_4_), and leave at room temperature. After centrifugation, the supernatant was discarded, immersed in the mixture of resin and acetone, and placed for 1 h at room temperature. After centrifugation, the supernatant was discarded and immersed in a mixture of resin and acetone. After centrifugation, the supernatant was discarded and soaked in 100% pure resin. It was embedded in pure resin and placed in an oven at 37℃. The polymerization was carried out at 60℃. The embedded sample was cut into 40 nm thick slices. The sections were placed on the copper net, and uranyl acetate dyeing solution was added drop by drop. The copper net was flipped so that the cutting side was downward, so that the sections floated on the staining solution, and the sections were left to stain. After staining, the sections were rinsed with distilled water e. Blot off excess water from slices and air dry. The sections were placed on the copper net, and lead citrate dye solution was continued to be added. The copper net was turned over again to make the cutting side close to the dye solution, and the sections were left to stain for 15 min. Finally, the ultrastructure of cytoskeleton was observed and photographed under transmission electron microscope (HITACHI, Japan) at 1.50 K, 8.00Kx magnification.

### Western blot analysis and cytoskeletal protein extraction

Total cell protein was extracted using cell protein lysate (Beyotime Biotechnology, China, Cat. no. P0013). Cell cytoskeletal proteins were extracted using Subcellualr Structure Protein Extraction Kit (Sangon Biotech, China, Cat. no. C500073) according to the instructions. After boiling protein samples in 5 × loading buffer (Beyotime Biotechnology, China, Cat. no. P0015), samples were separated on SDS-PAGE gels. The isolated protein is transferred to the PVDF membrane (Merck, Germany, Cat. no. ISEQ00010). PVDF membranes were blocked with 5% skim milk for 2 h and then assayed with indicated primary antibodies, including Ki-67 (Abcam, UK, Cat. no. ab16667), PCNA, Bcl-2, Bax, Cleaved-Caspase3 (CST, USA, Cat. no. 13110, Cat. no. 4223, Cat. no. 41162, Cat. no.9661), PTBP1 (Invitrogen, USA, Cat. no. 32-4800), Actin Reorganization Antibody Sampler Kit (CST, USA, Cat. no. 9967T), Paxillin and β-actin (Abcam, UK, Cat. no. ab32084, Cat. no. ab8227). The membranes were incubated with Anti-rabbit IgG, HRP-linked Antibody (CST, USA, Cat. no. 7074P2) and Anti-mouse IgG, HRP-linked Antibody (CST, USA, Cat. no. 7076P2) for 2 h at room temperature. Finally, the membranes were incubated using super sensitive ECL chemiluminescence kit (NCM Biotech, China, Cat. no. P10100). Blots were visualized with the Gel Doc XR^+^ system (Bio-Rad, USA).

### Immunofluorescence

Grow cells in 6-well plates containing confocal slides (1 × 10^4^ cells per well). After washing with PBS, cells were fixed with 4% paraformaldehyde for 15 min, and 0.5%Triton X-100 was permeable for 20 min. After the slides were soaked with PBS, 1%bovine serum albumin was blocked for 30 min, each slides were dripped with sufficient amount of diluted Paxillin antibody (Abcam, UK, Cat. no. ab32084) and incubated overnight at 4℃ in a wet box. Goat Anti-Rabbit IgG H&L (Alexa Fluor® 488) antibody (Abcam, UK, Cat. no. ab150077) was added in the dark and incubated in a wet box at 37℃. Then, cells were incubated overnight at 4℃ in a wet box with diluted PTBP1 antibody (Invitrogen, USA, Cat. no. 32-4800), followed by Goat Anti-Mouse IgG H&L (Alexa Fluor® 647) antibody (Abcam, UK, Cat. no. ab150115). Hoechst 33342 (Beyotime Biotechnology, China, Cat. no. C1025) was added to the drops and incubated for 5 min, and the specimens were nucleated. The tablets were sealed with a sealing solution containing an anti-fluorescence quench agent, and the acquired images were observed under a confocal laser scanning microscope (Carl Zeiss, Germany) at 200x magnification.

### PTBP1 Cas9-KO mouse model

The construction scheme of PTBP1 Cas9-KO Mouse Model was provided and constructed by Gempharmatech Co., Ltd (Nanjing, China). The concrete construction experiment scheme and verification scheme were presented in Supplementary File 1, 2. Briefly, the PTBP1 gene was modified by CRISPR/Cas9 technology. According to the structure of PTBP1 gene, exon 2 of PTBP1-216 (ENSMUST00000172282.7) was used as the knockout region. This region contains 31 bp of coding sequence, and knockdown of this region results in disruption of protein function. SgRNA is transcribed in vitro. Cas9 and sgRNA were injected into fertilized eggs of C57BL/6J mice. F0 positive mice were obtained by transplantation of fertilized eggs and confirmed by PCR and sequencing. F0 positive mice were mated with C57BL/6J mice to obtain a stable F1 generation mouse model.

### Xenograft assays and animal living imaging technology

Experimental mice (BALB/c nude mice and C57BL/6J mice) were normally fed until 5 weeks of age. MKN-28 in logarithmic growth phase (GC cells with red fluorescent label) were prepared into a cell suspension of 1.5 × 10^7^mL^−1^ with normal saline, and Matrigel (Corning, USA, Cat. no. 354262) was added at a ratio of 1:8 and mixed. The experimental mice were divided into Wild Type (WT) group and PTBP1 Cas9-KO group (For Nude mice xenograft model, the mice were divided into shNC group and shPTBP1 group.). Under sterile conditions, 0.2 ml of cell suspension was taken, the skin was lifted and slowly injected into the needle at a 15-degree Angle, and the needle was injected subcutaneously into the right anterior armpit of the experimental rats until the humiculus was raised. The tumor formation of the transplanted tumor was observed every 7 days. On day 21, Animal Living Imaging technology (PerkinElmer, USA) was used to dynamically observe the changes of fluorescence expression of GC cells in vivo. After 21 days, the mice were sacrificed, photographed, and the tumors were weighed and recorded.

### Immunohistochemistry

Using an immunohistochemical kit (Absin, China, Cat. no. abs996), the subcutaneous graft tumors were first immobilized with paraformaldehyde, followed by paraffin and frozen sections. Dewaxed and hydrated tissue sections were washed with PBST for 2–3 times for 5 min each, and antigenic repair was performed according to the requirements of primary antibody. Add 100µL endogenous peroxidase sealer and incubate for 10 min. PBST was washed with 100µL 10% goat serum and incubated for 20 min. Primary antibody Ki-67 (Abcam, UK, Cat. no. ab16667), F-actin (Abcam, UK, Cat. no. ab205) were added. 100µL HRP enzymo-labeled anti-mouse and rabbit secondary antibody polymer was added and incubated for 30 min, then freshly prepared DAB color developing solution was added and incubated for 5 min. 100µL hematoxylin was added and rinsed. Finally dehydrated, transparent and sealed. Images were acquired under an Orthostatic microscope (Olympus, Japan), at 40x magnification.

### Tissue microarray of GC

The tissue microarray of GC patients used in this experiment were provided by Shanghai Outdo Biotech CO., LTD. (Cat. no. HStmA180Su17). The tissue microarray contained 95 cases of survival gastric adenocarcinoma − 95 cancerous spots / 85 paracancer spots. The patient’s specific case is described in Supplementary File 3. For the multi-immunofluorescence experiment, put the tissue microarray in the oven and bake the wax for one hour. Complete in automatic dyeing machine. Antigen repair was done in microwave oven. The slides were removed and placed in a wet box. The slides were treated with H_2_O_2_ for 10 min. Place in a wet box, add blocking buffer and incubate. Remove blocking buffer, drop diluted primary antibody working solution, including PTBP1 (Invitrogen, USA, Cat. no. 32-4800) and CK (Abcarta, China, Cat. no. PA125), TBST wash slides, drop secondary antibody, incubate for 10 min. Opal dye diluent was added (PerkinElmer, USA, Cat. no. NEL801001KT), incubated at room temperature for 10 min, and TBST was used to clean the slides. Re-dye subsequent indicators and repeat steps until all indicators are marked. DAPI working solution was added and incubated at room temperature for 5 min. TBST cleaned the slides, removed the slides, and fluorescent anti-quenching tablet was added to seal the slides. The automatic quantitative pathological imaging system (TissueGnostics, Austria) was used to photograph and observe at 2.5, 10x magnification. TissueFAXS Viewer software (TissueGnostics, Austria) was used for cell differentiation and statistics, and cell numbers of different cells were counted separately to obtain their distribution in the tumor.

### Bioinformatic analysis

The difference of PTBP1 expression between normal people and GC patients was obtained by analyzing TCGA database using Gene Expression Profiling Interactive Analysis (GEPIA) (http://gepia2.cancer-pku.cn/#index) [10]. The survival curve of PTBP1 in TCGA-STAD was generated by Kaplan-Meier Plotter (https://kmplot.com/analysis/index.php?p=service&cancer=gastric) [11]. 10 genes (EZR, RDX, MSN, CDH1, CDH2, VIM, CD44, MMP9, MMP14, TGFB1) is thought to be involved in the cell actin cytoskeleton remodeling [12, 13] and epithelial mesenchymal transformation (EMT) [14] process. We put these 10 genes into GEPIA as signatures and calculated the correlation with PTBP1.

### Statistical analysis

Data are presented as the means ± SEM. Student’s *t* test or One-way analysis of variance (ANOVA) with Tukey’s or Dunnett’s post‑hoc test. *p*-values less than 0.05 were considered statistically significant (* *p* < 0.05, ** *p* < 0.01, and *** *p* < 0.001). Data analysis was performed using GraphPad Prism 7 (GraphPad Software, USA) and SPSS 24.0 (IBM, USA).

## Results

### PTBP1 is highly expressed in GC patients and is related to actin skeleton remodeling and EMT signaling pathways

**Fig. 1:**
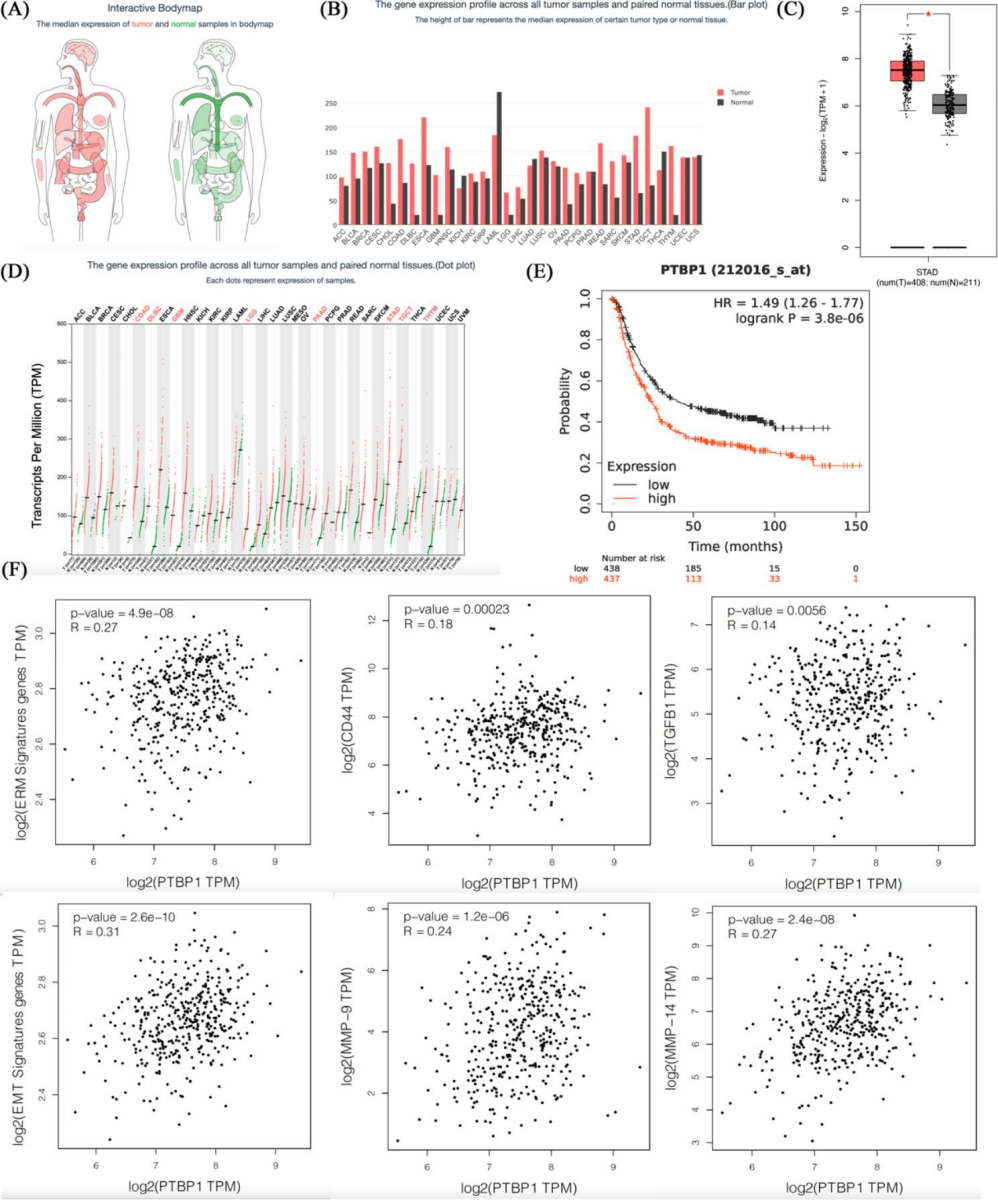
(**A, B, C**) The expression of PTBP1 in normal people and GC patients, *n* = 619. Graphs were obtained from GEPIA. (**D**) The gene expression profile across all tumor samples and paired normal tissues. Each dots represent expression of samples. Graphs were obtained from GEPIA. (**E**) Kaplan–Meier analysis of overall survival curve in GC patients with high PTBP1 expression versus low PTBP1 expression, *n* = 875. Graphs were obtained from Kaplan-Meier Plotter. (**F**) Correlation between EMT related genes or actin skeleton remodeling related genes and PTBP1 was generated from GEPIA. Pearson correlation. Graphs were obtained from GEPIA. * *p* < 0.05, ** *p* < 0.01 and *** *p* < 0.001

GEPIA was used for bioinformatics analysis. Firstly, we analyzed the expression of PTBP1 in normal people and GC patients. Using TCGA database, the results showed that PTBP1 was highly expressed in GC patients compared with normal people (Fig. 1A, B, C, * *p* < 0.05). At the same time, PTBP1 was highly expressed in GC patients compared with other tumor types (Fig. 1D). The survival curve of PTBP1 in TCGA-STAD was generated by Kaplan-Meier Plotter (Fig. 1E, *** *p* < 0.001). We put these 10 genes (EZR, RDX, MSN, CDH1, CDH2, VIM, CD44, MMP9, MMP14, TGFB1) into GEPIA as signatures and calculated the correlation with PTBP1. The results showed that PTBP1 was correlated with EMT related genes and actin skeleton remodeling related genes (Fig. 1F, *** *p* < 0.001).

### Knockdown PTBP1 in GC cells inhibited proliferation

**Fig. 2:**
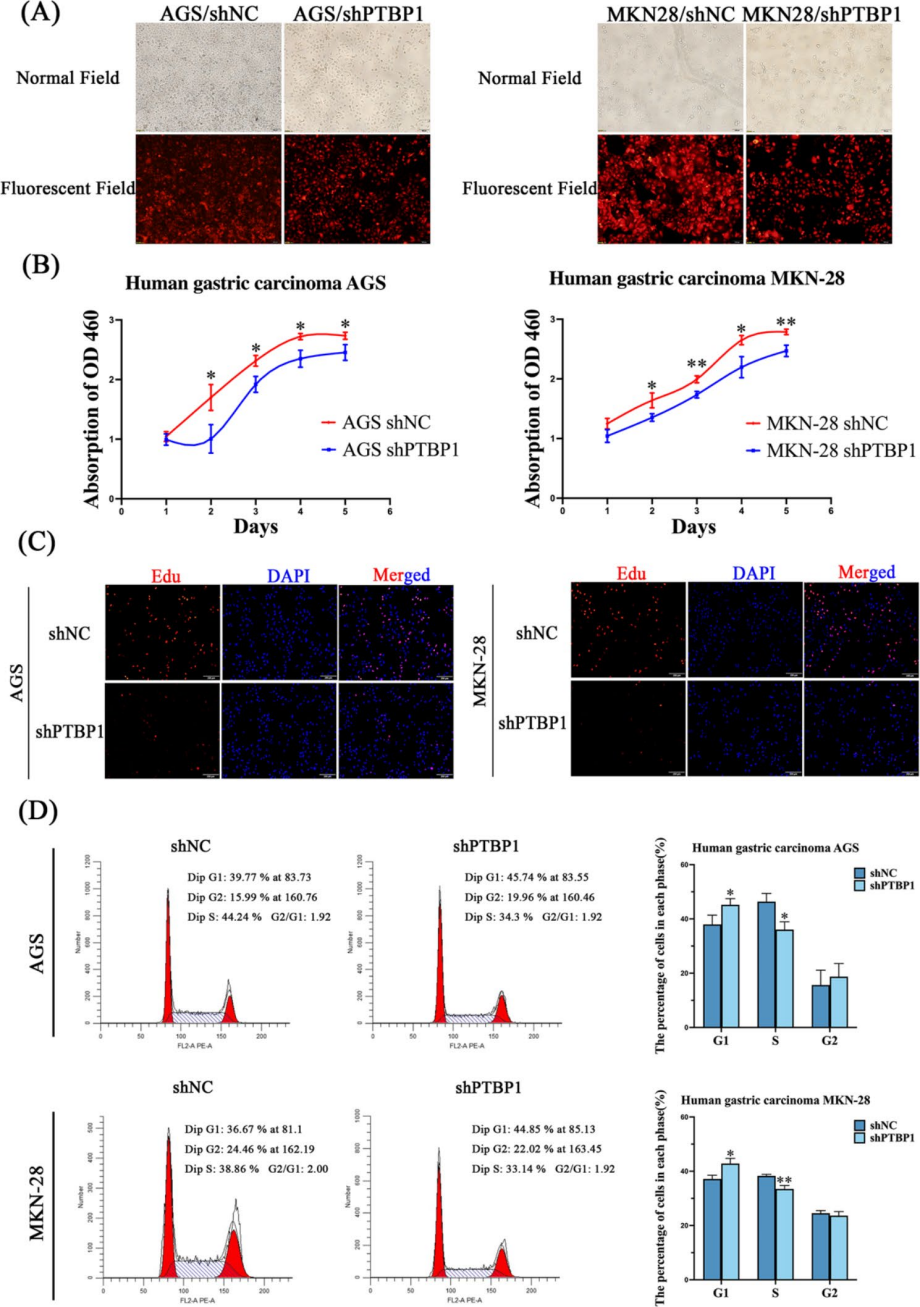
(**A**) The transfection efficiency of two GC cell lines was observed by fluorescence inverted microscope (20x magnification, scale bar: 100 μm). (**B**) CCK8 assay showed that the proliferation rate of GC cells in shPTBP1 group was decreased compared with that in shNC group over time. (**C**) EdU staining of GC cells in shPTBP1 group was significantly weaker than that in shNC group (10x magnification, scale bar: 200 μm). (**D**) The cell cycle was detected using a cell cycle detection kit. The results showed that the number of G1-phase cells in shPTBP1 group was increased compared with that in shNC group, while the number of cells in S phase decreased. * *p* < 0.05, ** *p* < 0.01

To test whether PTBP1 has an effect on the proliferation of GC cells, we successfully knocked down PTBP1 in two GC cell lines by transfection with a designed lentivirus (Fig. 2A). EdU assay and Cell Counting Kit-8 (CCK-8) assay were used to verify that the cell proliferation was inhibited after PTBP1 knockdown (Fig. 2B, C, * *p* < 0.05, ** *p* < 0.01). The results of cell cycle detection showed that the number of G1-phase cells in shPTBP1 group was increased compared with that in shNC group, while the number of cells in S phase decreased. This indicates that the cells were arrested in G1-phase (Fig. 2D, * *p* < 0.05, ** *p* < 0.01). This suggests that PTBP1 plays an important role in the proliferation of GC cells.

### Knockdown PTBP1 in GC cells inhibited cell cloning ability and wound healing ability

**Fig. 3:**
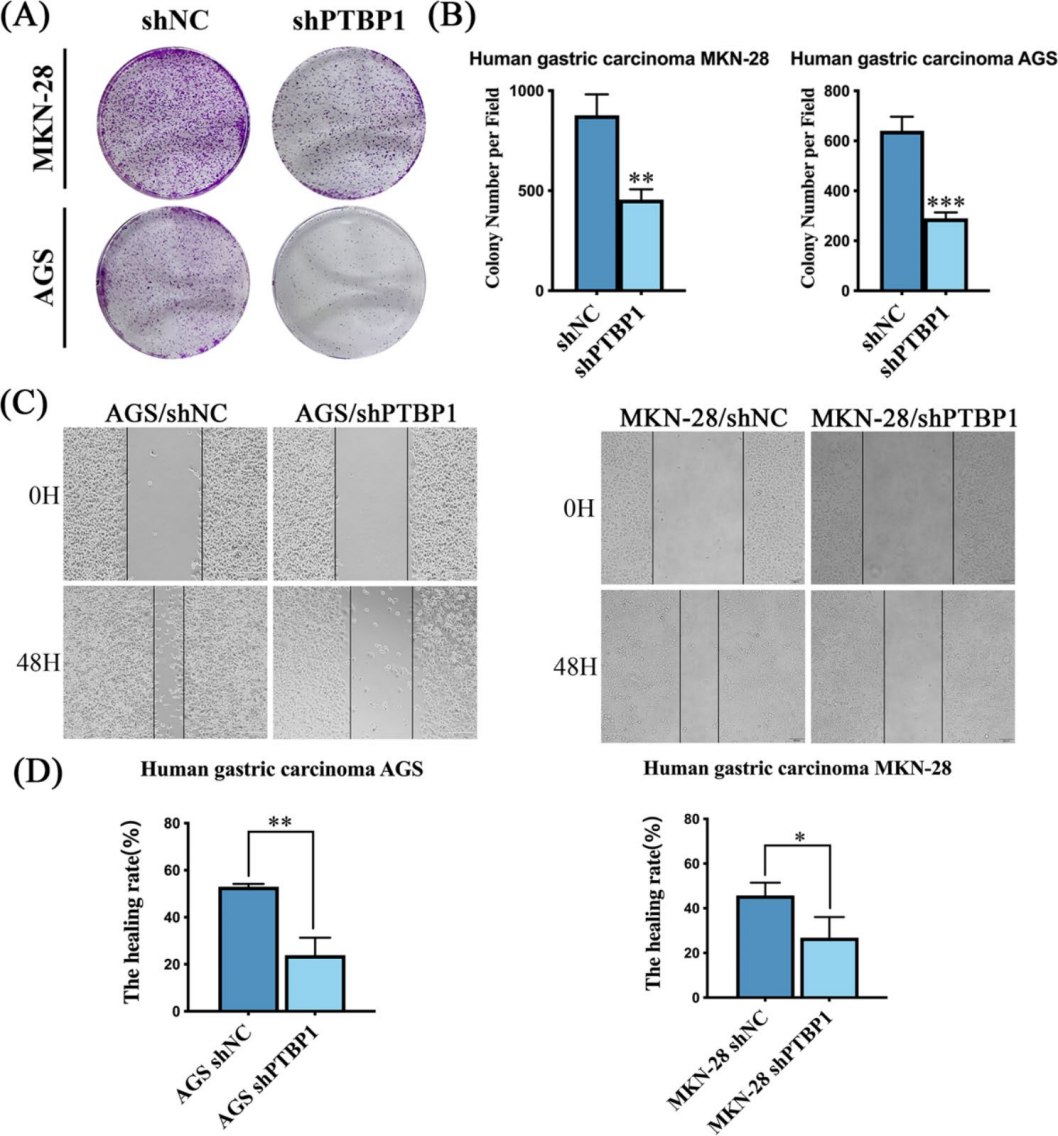
(**A, B**) Cell cloning and formation assay was performed to detect cell proliferation ability. Cells were fixed with paraformaldehyde and stained with crystal violet solution. Image J software was used for cell count. (**C, D**) The wound healing test results showed that the wound healing ability of the two GC cells was inhibited after PTBP1 knockdown (20x magnification, scale bar: 100 μm). * *p* < 0.05, ** *p* < 0.01 and ***p<0.001

Clone formation assay was used to detect the cloning ability of two GC cell lines. The results showed that after PTBP1 knockdown, the clone formation ability of the cells was weakened, indicating that the proliferation ability of the cells was inhibited (Fig. 3A, B ** *p* < 0.01, ***p<0.001). The wound healing ability of GC cells was tested by wound-healing assay. The results showed that the wound-healing ability of GC cells was weakened after PTBP1 knockdown (Fig. 3C, D * *p* < 0.05, ** *p* < 0.01).

### The division and proliferation ability of GC cells were observed dynamically after knockdown PTBP1

**Fig. 4:**
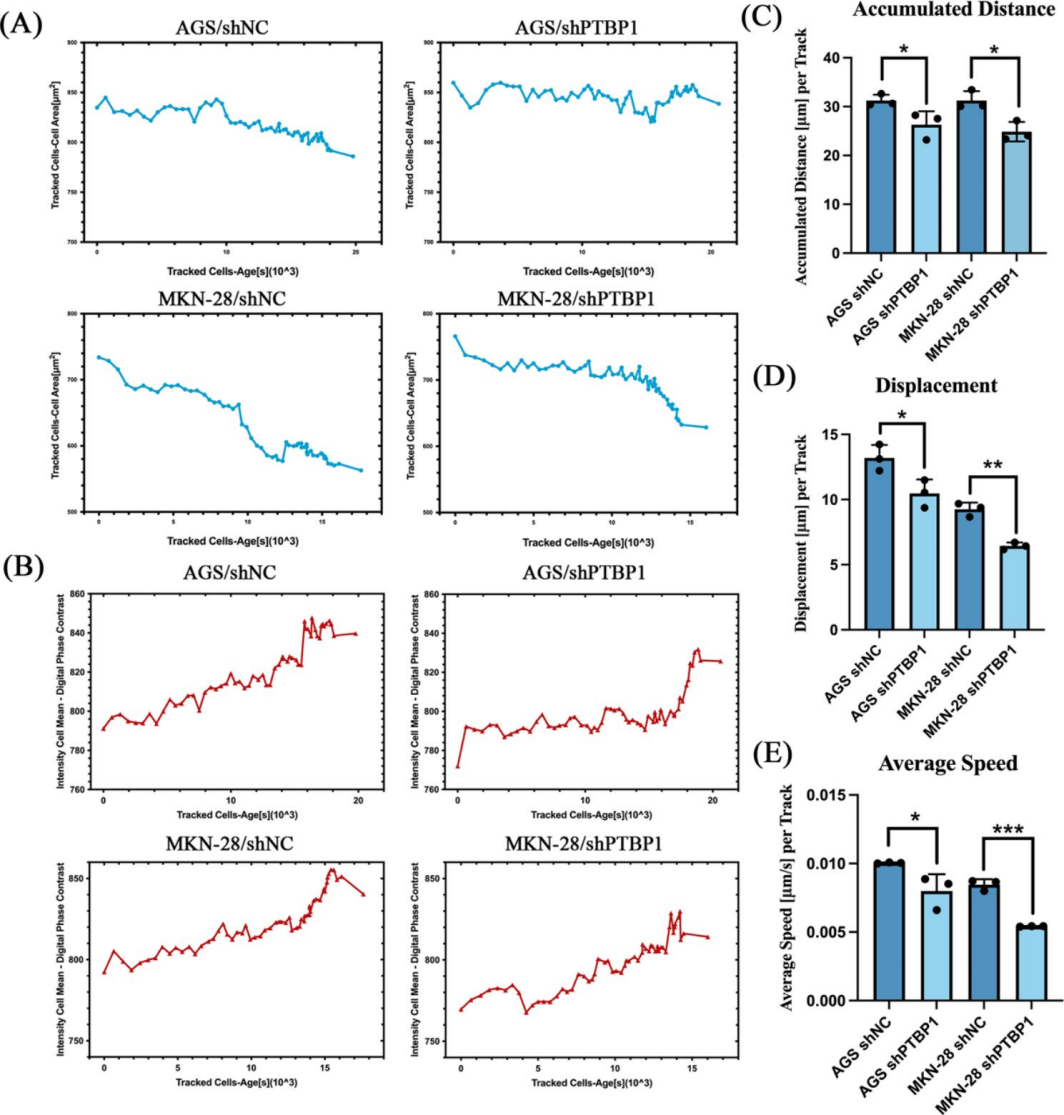
(**A**) Numerical data from the evaluation provided was used to visualize characteristics of dividing cells. To generate the plots, the track results table was sorted by start type and end type. Only cells with start and end type *Split* were displayed. Grouping was set to *Object*. Cell area or (**B**) mean intensity DPC were plotted against cell age. Tracked Cells-Age[s]: Time passed since the cell was first observed in seconds; Tracked Cells-Cell Area[µm^2^]: Average cell size over all cells per well; Intensity Cell Mean -Digital Phase Contrast: Intensity DPC signal, first averaged over all pixels per cell and then averaged over all cells per well. (**C**) Total length of all segments of an object track in µm; averaged over all tracks per well. (**D**) Distance from the first to last observation of a cell track in µm; averaged over all tracks per well. (**E**) Accumulated distance in µm divided by the track duration in seconds; calculated per track and then averaged over all tracks per well. *p < 0.05, ** *p* < 0.01 and *** *p* < 0.001

To dynamically observe the division and proliferation of GC cells, we used Operetta CLS high-content cell imager HICIS. Image segmentation is performed using the building block find Cells-Method P. Method P is dedicated to the segmentation of digital phase contrast (DPC) images. Parameters have been adjusted to aim for robust object segmentation rather than for precise detection of cell outlines. Object tracking is performed by the building block Track Objects. To evaluate the outcome of object tracking on the image analysis screen, a time window has to be selected. Note how cells increase in cell area over the cell cycle progression. Cell rounding during mitosis is reflected by a pronounced decrease in cell size and a strong increase in the DPC signal. As can be seen from the figure, when PTBP1 was knocked down, the cell areas increased (Fig. 4A) and the mean intensity DPC decreased (Fig. 4B). In addition, we dynamically tracked the cell movement trajectory and analyzed Accumulated Distance [µm] per Track (Fig. 4C * *p* < 0.05), Displacement [µm] per Track (Fig. 4D * *p* < 0.05, ** *p* < 0.01) and Average Speed [µm/s] per Track (Fig. 4E * *p* < 0.05, *** *p* < 0.001), and found that the motility of GC cells was inhibited after PTBP1 knockdown. This means that the cell’s ability to divide and proliferate is reduced.

### Knockdown PTBP1 in GC cells inhibited actin cytoskeleton remodeling

**Fig. 5:**
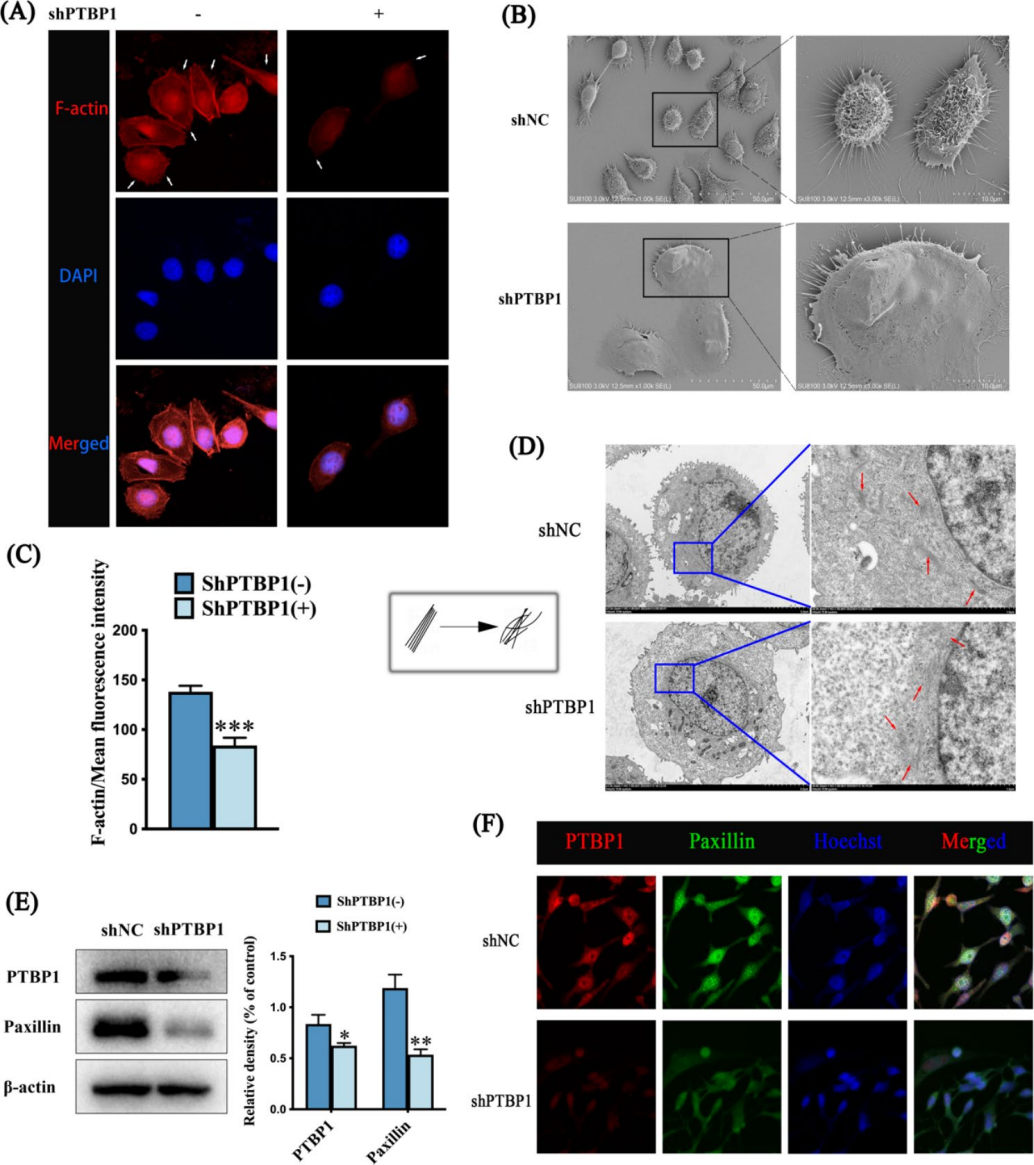
(**A**) The white arrows indicate pseudopodia around the cells. Images of cytoskeletal staining were acquired with a confocal laser scanning microscope (1000x magnification, scale bar: 10 μm). (**B**) Scanning electron microscopy was used to observe the cell morphology. Compared with shNC group, the cell morphology and cell pseudopodia of shPTBP1 group were changed. Images were captured by scanning electron microscopy (1.00 K, 3.00Kx magnification, scale bar: 50 μm and 10 μm). (**C**) Image J software was used to analyze the mean fluorescence intensity of F-actin. (**D**) Transmission electron microscopy was used to observe GC cells. Compared with shNC group, microfilaments in shPTBP1 group changed from neatly arranged to disordered, indicating that actin skeleton remodeling was inhibited. On the left is a simple diagram of microfilament changes. The red arrows on the right indicate microfilaments in cells. Images were captured by transmission electron microscopy (1.50 K, 8.00Kx magnification, scale bar: 5 μm and 1 μm). (**E**) Western blot was used to detect the expression levels of PTBP1 and Paxillin in GC cells. (**F**) Immunofluorescence assays were conducted to detect the expression of PTBP1 and Paxillin in GC cells with PTBP1knockdown (200x magnification, scale bar: 50 μm). *p<0.05, ** *p* < 0.01 and ***p<0.001

To observe the effect of PTBP1 on the remodeling of the actin skeleton in GC cells, we first stained the cytoskeleton. The results showed that cytoskeleton staining (F-actin) fluorescence decreased and the number of pseudopodia around the cells decreased (Fig. 5A, C *** *p* < 0.001). Image J software was used to analyze the mean fluorescence intensity of F-actin. Then we observed the morphology of GC cells by SEM. The results showed that the morphology of GC cells was significantly changed after PTBP1 knockdown. Most of the cells in shNC group had long filopodia around the cells, while shPTBP1 group had fewer filopodia on the cell surface, with a few large sheet pseudopodia (Fig. 5B). Scanning electron microscope (HITACHI, Japan) at 1.00 K, 3.00Kx magnification, photography. To further verify the effect of PTBP1 on the cytoskeleton, the changes of cell microstructure were observed by TEM. The results showed that compared with GC in shNC group, the number of microfilaments in shPTBP1 was relatively small, and the microfilaments in shPTBP1 group were changed from neatly arranged microfilaments to disordered microfilaments, which indicated that the remodeling of cellular actin skeleton was affected (Fig. 5D). Transmission electron microscope (HITACHI, Japan) at 1.50 K, 8.00Kx magnification, photography. Cell adhesion is a key step in the remodeling of actin skeleton leading to cell movement. We then used Western blot assay and immunofluorescence assay to detect the adhesion marker protein paxillin. The results showed that when PTBP1 was knocked down, the protein expression (Fig. 5E * *p* < 0.05, ** *p* < 0.01) and fluorescence expression (Fig. 5F) of paxillin were decreased. Confocal laser scanning microscopy (Carl Zeiss, Germany) was used to obtain the images at 200x magnification, photography. This also confirmed that PTBP1 is involved in cellular actin skeleton remodeling.

### PTBP1 may regulate the expression of proliferation signaling pathway and actin skeleton remodeling signaling pathway in GC cells

**Fig. 6:**
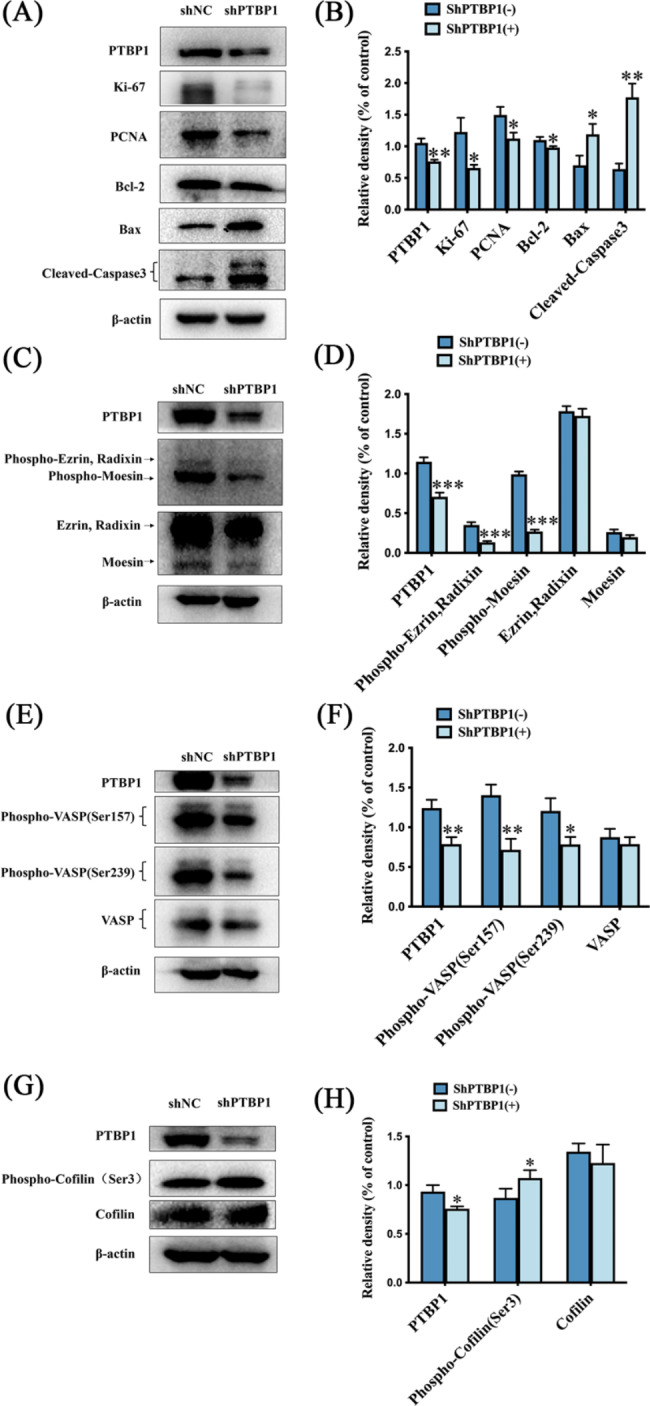
(**A, B**) Western blot was used to detect the expression levels of PTBP1, Ki-67, PCNA, Bcl-2, Bax and Cleaved-Caspase3 in GC cells. (**C, D**) Western blot was used to detect the expression levels of PTBP1, phospho-ezrin, phospho-Radixin, phospho-Moesin, Ezrin, Radixin and Moesin in GC cells. (**E, F**) Western blot was used to detect the expression levels of PTBP1, phospho-VASP (Ser157), phospho-VASP (Ser239) and VASP in GC cells. (**G, H**) Western blot was used to detect the expression levels of PTBP1, phospho-cofilin (Ser3) and Cofilin in GC cells. * *p* < 0.05, ** *p* < 0.01 and *** *p* < 0.001

Total cell proteins were extracted, and then the expression levels of proliferation markers and apoptosis-related signaling pathways were detected by Western blot. Cytoskeletal protein was extracted from GC cells using cytoskeletal protein extraction kit. Subsequently, Western blot was used to detect the changes of signaling pathways related to actin skeleton remodeling. As shown in Fig. 6, the protein expression of Ki-67, PCNA and Bcl-2 decreased, while the expression of Bax and Cleaved-Caspase3 proteins increased (Fig. 6A, B, * *p* < 0.05, ** *p* < 0.01). The protein expression of Phospho-Ezrin, Radixin and Phospho-Moesin decreased (Fig. 6C, D, *** *p* < 0.001). The protein expression of Phospho-VASP(Ser157) and Phospho-VASP(Ser239) decreased (Fig. 6E, F, * *p* < 0.05, ** *p* < 0.01). The protein expression of phospho-cofilin (Ser3) increased (Fig. 6G, H, * *p* < 0.05).

### PTBP1 regulates the proliferation of xenograft GC cells in vivo

**Fig. 7:**
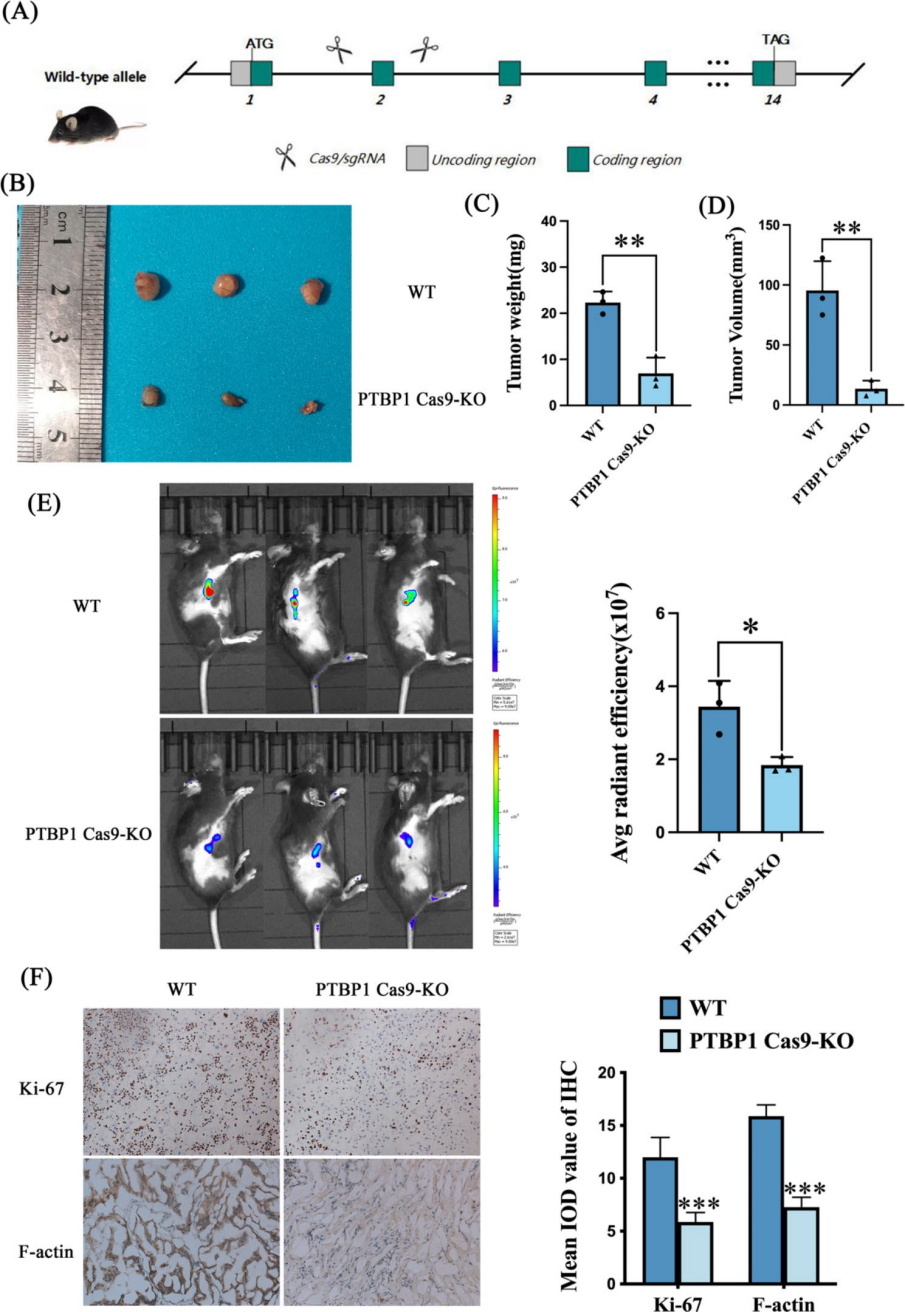
(**A**) Brief schematic of PTBP1 Cas9-KO mouse model construction. (**B**) After 21 days, the mice were sacrificed, photographed, and the tumors were weighed and recorded. (**C, D**) Weight and volume statistics of subcutaneous xenografts. (**E**) Observed the fluorescence intensity of subcutaneous xenografts using animal living imaging. The fluorescence intensity scale of the WT group ranged from 5.8 × 10^7^ to 9 × 10^7^, while that of the PTBP1 Cas9-KO group ranged from 2.8 × 10^7^ to 8.8 × 10^7^. (**F**) Immunohistochemical techniques were used to detect the expression of Ki-67 and F-actin in subcutaneous graft tumors (40x magnification, scale bar: 50 μm). * *p* < 0.05, ** *p* < 0.01 and *** *p* < 0.001

The results of subcutaneous xenograft tumor experiments in nude mice showed that when PTBP1 was knocked down, the growth of subcutaneous xenograft tumors was inhibited (Supplementary File 4-Figure S1, *** *p* < 0.001). On this basis, in order to further clarify the role of PTBP1 in vivo, we successfully constructed the PTBP1 Cas9-KO mouse model, and observed the fluorescence intensity of subcutaneous xenografts using animal living imaging. After 21 days, the mice were sacrificed, photographed, and the tumors were weighed and recorded. The results showed that the volume and weight of subcutaneous xenografts in PTBP1 Cas9-KO group were smaller and lighter than those in WT group (Fig. 7B, C, D, ** *p* < 0.01). The results showed that the fluorescence intensity of subcutaneous xenografts in PTBP1 Cas9-KO group was weaker than that in WT group (Fig. 7E, * *p* < 0.05). Immunohistochemical experiments on the peeled subcutaneous grafts showed that the expression of the proliferative marker Ki-67 in the PTBP1 Cas9-KO group was lower than that in the WT group. At the same time, the skeleton marker protein F-actin was lower in PTBP1 Cas9-KO group (Fig. 7F, *** *p* < 0.001). Images were acquired under an Orthostatic microscope (Olympus, Japan).

### PTBP1 is highly expressed in patients with GC and is associated with poor prognosis

**Fig. 8:**
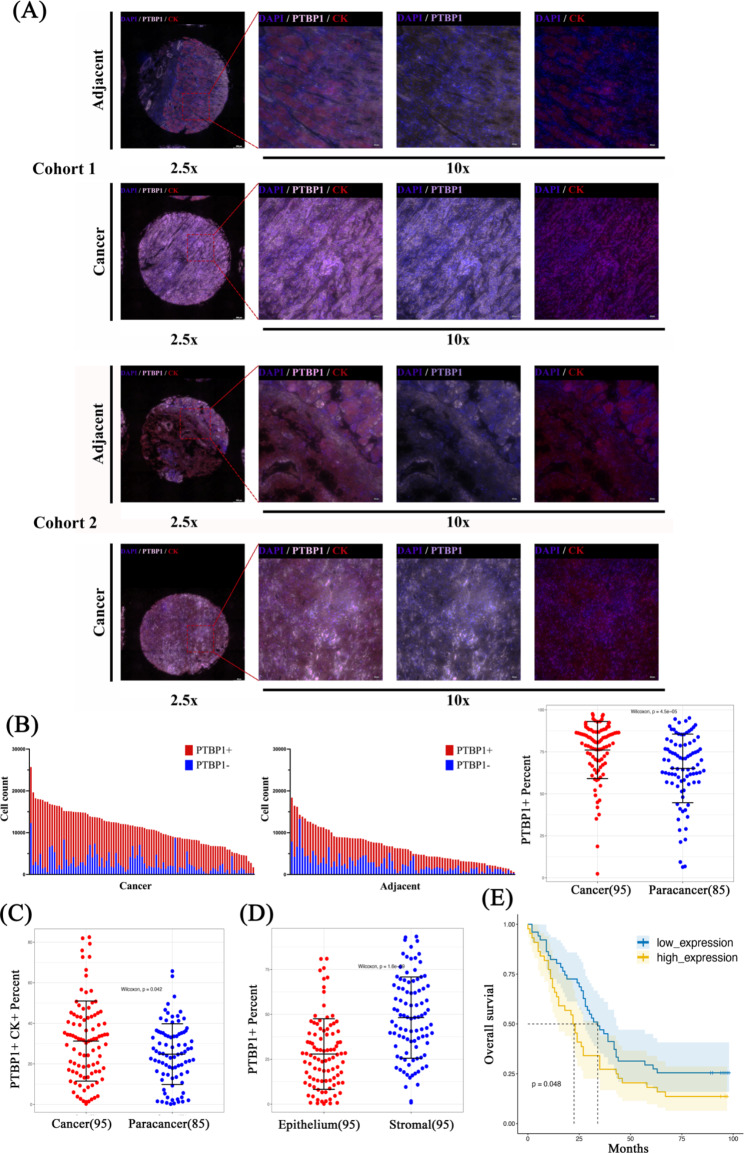
(**A**) Multiple immunofluorescence technology was used to stain GC tissue microarray, and automatic quantitative pathological imaging system was used to photograph, observe and quantitatively analyze (2.5, 10x magnification, scale bar: 200 μm and 50 μm). (**B**) The number of different cells was counted separately to obtain their distribution in the tumor, and then the differential expression of the whole tissue cancer and the paracancer was analyzed. (**C**) The differential expression of whole tissue carcinoma and paracancer was analyzed under the condition of positive PTBP1 and CK double staining. (**D**) The number of different cells were counted to obtain their distribution in the tumor, and then the differences in epithelium and stromal expression were analyzed. (**E**) Univariate overall survival was analyzed using Kaplan-Meier survival analysis and log-rank statistical test. * *p* < 0.05, *** *p* < 0.001

To detect the expression of PTBP1 in patients with GC, we used GC tissue microarray (95 cases of survival gastric adenocarcinoma, including 95 cancer sites / 85 paracancer sites) for multiple immunofluorescence staining of PTBP1 and CK (CK staining was used to distinguish epithelial tissues). The patient’s specific case is described in Supplementary File 3. The automatic quantitative pathological imaging system (TissueGnostics, Austria) was used to photograph and observe at 2.5, 10x magnification. TissueFAXS Viewer software (TissueGnostics, Austria) was used for cell differentiation and statistics, and cell numbers of different cells were counted separately to obtain their distribution in the tumor. The results showed that compared with the adjacent tissues, the fluorescence expression of PTBP1 in the cancer tissues was not only higher (Fig. 8A), but also the percentage of PTBP1 labeled cells was higher, suggesting that PTBP1 was highly expressed in patients with GC (Fig. 8B *** *p* < 0.001). In addition, the proportion of PTBP1/CK double-label staining positive cells was much higher than that of paracancer tissue. This suggests that PTBP1 is highly expressed in the epithelial tissues of patients with GC (Fig. 8C *** *p* < 0.001). We also statistically investigated the expression of PTBP1 in the epithelium and stromal cells of GC tissue, and found that PTBP1 was highly expressed in the stromal cells (Fig. 8D *** *p* < 0.001). Finally, combined with the patient’s medical records, Kaplan-Meier survival analysis and log-rank statistical test were used for univariate analysis of survival. The results showed that high expression of PTBP1 was closely related to poor prognosis in patients with GC (Fig. 8E * *p* < 0.05). These results all indicate that PTBP1 is highly expressed in patients with GC and is closely associated with poor prognosis. In addition, univariate and multivariate Cox regression analysis showed that PTBP1 was an independent factor affecting the prognosis of GC. GC patients with high PTBP1 expression usually have a poor prognosis (Table 1). This suggests that PTBP1 may be a new target for the clinical treatment of GC.

**Table 1:** Univariate and multivariate analysis of the factors correlated with the overall survival of GC patients

variables	Univariate analysis				Multivariate analysis			
	HR	95%CI		*p* value	HR	95%CI		*p* value
		Lower limits	Upper limits			Lower limits	Upper limits	
**PTBP1**	3.238	1.203	8.713	***0.020***	2.714	1.023	7.204	***0.045***
**Sex**	1.432	0.855	2.396	0.172				
**Age**	1.215	0.775	1.905	0.397				
**Grade stage**	2.216	1.255	3.913	***0.006***	2.15	1.16	3.97	***0.015***
**Tumor size**	2.201	1.383	3.501	***0.001***	1.7	1.05	2.77	***0.032***
**T stage**	3.446	1.382	8.594	***0.008***	4.08	1.38	12.06	***0.011***
** N stage**	2.87	1.467	5.613	***0.002***	2.69	1.03	7.02	***0.043***
**TNM stage**	2.496	1.489	4.185	***0.001***	0.8	0.38	1.7	0.565

Univariate and multivariate cox regression analysis. Variables with a *p* value less than 0.05 in univariate analysis were incorporated into the cox multivariate analysis, and if the *p* value was still smaller than 0.05, this variable could be used as an independent factor affecting the prognosis. *p* < 0.05 was statistically significant

## Discussion

At present, surgery is considered as the first choice for the early treatment of GC. However, most patients are already advanced at the time of diagnosis and miss the best opportunity for surgery. Patients with advanced GC usually adopt the combination of adjuvant chemoradiotherapy, molecular targeted therapy and immunotherapy to prolong the survival time [15]. Most deaths are due to the lack of effective antitumor therapy [16]. It is urgent to find new therapeutic targets and provide new therapeutic strategies for GC. Tumor molecular targeted therapy has the advantages of high selection, well-targeted, easy to be accepted by patients, and small response to local or systemic therapy, which can ultimately achieve the goal of effective tumor control and reduce the damage of normal tissues around tumors. It’s a promising treatment method.

The cytoskeleton is composed of reticulin fibers, which are closely related to cell shape and cell transport. Studies have found that mechanical forces in cells can affect the assembly of the cytoskeleton, and thus affect cell proliferation [17]. Control the mechanical force of cytoskeleton proteins can be induced activation that promote tumor growth factor, when cytoskeletal dynamics change will cause protein reticular fibers in different degrees of rearrangement of the cell proliferation and tissue overgrowth have direct effect, if the elasticity of the cytoskeleton would rapidly reduce cell proliferation [18, 19]. According to their diameters, the cytoskeleton is mainly divided into Microtubule, Microfilament and Intermediate filament [5]. Actin is the main component of microfilaments and plays a key role in maintaining cell morphology and movement [20]. During tumor cell movement, actin-rich protrusions formed by dynamic remodeling of the actin cytoskeleton are the structural basis for tumor cells to perform invasion and migration functions [21]. Among them, tumor cell pseudopodia mainly include Invadopodia, Lamellipodia and Filopodia [22]. Therefore, researching the regulatory mechanism of actin cytoskeleton remodeling in pseudopodia formation may provide a feasible therapeutic target for clinical prevention and treatment of tumor invasion and metastasis.

PTBP1 can be involved in the regulation of mRNA processing, such as polyadenylation, nucleoplasmic shuttling, mRNA stability and mRNA translation [23–26]. Recent studies have shown that PTBP1 is involved in the occurrence and development of a variety of tumors, but the specific mechanism of how PTBP1 regulates the occurrence and development of tumors in GC has not been clarified. To explore whether PTBP1 is associated with GC, we first used bioinformatics analysis and found that PTBP1 was highly expressed in GC patients. It is closely related to poor prognosis and short survival time of GC patients. The clinical manifestations of middle and advanced GC are mostly accompanied by adjacent tissue or organ invasion, intraperitoneal lymph node metastasis and organ metastasis, and the prognosis is poor. How to inhibit the invasion and metastasis of GC has been the focus of research in recent years, in which cellular actin skeleton remodeling provides the impetus for the invasion and metastasis of GC. EMT is closely related to TGF-β, and the process of EMT involves actin skeleton remodeling, which is partly driven by the altered expression of ERM protein. ERM also interacts in part through its receptor function with CD44, a cell surface glycoprotein abundantly expressed in some cancer stem cells and closely associated with cell motility and cancer metastasis. Invasive pseudopodia can recruit the proteases MMP-9 and MMP-14 to the leading edge [27, 28], where they degrade ECM and basement membrane, thereby promoting tumor invasion and metastasis. Bioinformatics results showed that PTBP1 was closely related to EMT and actin skeleton remodeling signaling pathways. This suggests that PTBP1 is involved in the development, invasion and metastasis of GC.

We knocked down the PTBP1 gene in GC cells AGS and MKN28, and found that the division and proliferation ability of GC cells was inhibited after PTBP1 knockdown. The results of cell cycle detection showed that the number of G1-phase cells in shPTBP1 group was increased compared with that in shNC group, indicating that the cells were arrested in G1-phase. Scratch assay showed that the migration ability of PTBP1 was inhibited after PTBP1 knockdown. The high-content tracking system also confirmed that PTBP1 regulates GC cells division. These results suggest that PTBP1 may be involved in the proliferation of GC cells.

Whether PTBP1 regulates actin skeleton remodeling in GC remains unclear. The results of skeletal staining and SEM showed that pseudopodia decreased and cell morphology changed in GC cells with PTBP1 knockdown. Moreover, the results of TEM showed that the remodeling and rearrangement of microfilaments in GC cells were affected, which indicated that the remodeling of cellular actin skeleton was significantly inhibited. The process of cell migration includes the extension of lamellipodia at the front of the cell, the formation of focal adhesion (FA), and so on. FA is the contact point between cells and the surrounding environment, which can integrate intracellular and extracellular signals to regulate cell migration [29]. And for cell movement, adhesion is one of the factors. In the first step of migration, the lamellar pseudopods of the cells need to form adhesion to the substrate in order to have a point of support to pull the cells [30]. Therefore, Western blot and immunofluorescence techniques were used here to detect paxillin, a marker of FA, in order to explore whether PTBP1 can affect the growth of adhesive plaques. We hypothesized that the disturbance of microfilament rearrangement would affect cell mitosis and thus cell proliferation.

Therefore, we used Western blot assay to detect cell proliferation and apoptosis signaling pathways. Proliferating cell nuclear antigen (PCNA) is a member of the DNA sliding clamp family that aids in DNA replication [31]. Many proteins involved in DNA replication, DNA repair, and cell cycle control bind PCNA rather than directly to DNA, thereby facilitating rapid DNA processing [32]. PCNA protein expression is a widely recognized marker of proliferation. The results showed that the expression of PCNA in shPTBP1 group decreased, which was consistent with the previous cell cycle results, indicating that knockdown of PTBP1 could affect DNA synthesis of GC cells and thus affect cell proliferation. In addition to PCNA, Ki-67 is also an indicator of cell proliferation and a biomarker for predicting the prognosis of GC patients [33]. We found that knockdown of PTBP1 affected the protein expression level of Ki-67. In addition, the apoptosis signaling pathway was regulated [34, 35], and PTBP1 knockdown promoted the apoptosis of GC cells.

In addition, we also investigated the effect of PTBP1 on proteins related to cytoskeleton remodeling signaling pathway. Small protein filamentins are a conserved family of actin-binding proteins that promote actin filament regeneration by cutting the original filament [36]. Filamentins are phosphorylated at Ser3 by LIMK or TESK, and thus their filamentous activity is inhibited [37–39]. Ezrin, radixin and moesin (ERM) proteins are involved in cell adhesion, membrane edge fluctuations, and microvilli formation [40, 41]. Interacting cytosolic ERM proteins exist as monomers or dimers and can form intramolecular and intermolecular binding through their amino and carboxyl terminal domains [42]. Phosphorylation at carboxy-terminal threonine residues can alter protein conformation and disrupt binding of these proteins and lead to ERM protein activation [43, 44]. Vasodilator stimulated phosphoprotein (VASP) is an adaptor protein that links the cytoskeleton to signal transduction pathways and plays a role in fibroblast migration, platelet activation, and axonal guidance [45, 46]. Active VASP appears to promote actin polymerization by limiting the capping of actin filaments, with phosphorylation of PKA inhibiting this anti-capping activity [47–50]. Western blot results showed that the actin skeleton remodeling related signaling pathways were regulated after PTBP1 knockdown, and the cellular actin skeleton remodeling was inhibited.

In order to further investigate the effect and mechanism of PTBP1 on actin skeleton remodeling in GC cells in vivo, we successfully constructed PTBP1 Cas9-KO mouse model, which provides reliable research support for elucidating the related mechanism of GC treatment in the whole animal level. Animal Living Imaging System showed that the tumor fluorescence intensity of PTBP1 Cas9-KO group was weaker than that of WT group. Compared with WT group, PTBP1 Cas9-KO group had smaller tumor volume and weight. In addition, immunohistochemical experiments were performed on the exfoliated subcutaneous graft tumors, and the proliferative indicator Ki-67 and the skeleton-marker protein F-actin were detected. The results further demonstrated that PTBP1 could regulate actin skeleton remodeling and inhibit the proliferation of GC cells.

Finally, we further verified our conjecture in the clinical case samples of GC patients. We performed multiple immunofluorescence experiments on tissue microarray containing 95 cases of survival gastric adenocarcinoma, and found that PTBP1 was highly expressed in tissues of GC patients, and was closely associated with poor prognosis of GC patients. This is consistent with our bioinformatics predictions. This also confirmed the hypothesis that PTBP1 is a GC oncogene.

## Conclusions

In summary, PTBP1 may regulate the remodeling of actin skeleton in GC cells, thereby inhibiting the proliferation of GC cells and affecting the survival and prognosis of GC patients. PTBP1 has the biological function of alternative splicing, which can selectively splice Pre-mRNA, produce diversified mRNAs, transcriptional regulation of a variety of proteins, and mediate tumor cell survival, proliferation, metastasis and other processes [51, 52]. We hypothesized that PTBP1 may alternative splicing some actin skeleton remodeling genes, thereby regulating actin skeleton remodeling, which will be further verified in subsequent experiments. Nevertheless, we still believe that PTBP1 plays an important role in the occurrence and development of GC, and it has potential significance to use this gene in the diagnosis and treatment of GC.

### Electronic supplementary material

Below is the link to the electronic supplementary material.

**Supplementary File 1**: Genotyping Report

**Supplementary File 2**: Ptbp1 Cas9-KO Strategy

**Supplementary File 3**: Gastric cancer tissue microarray HStmA180Su17 cases and array layout table

**Supplementary File 4**: Figure S1

## Data Availability

The original contributions presented in the study are included in the article/Supplementary files. Further inquiries can be directed to the corresponding author.

## Abbreviations

PTBP1: Polypyrimidine tract binding protein 1
GC: Gastric cancer
CCK-8: Cell counting kit-8
DPC: Digital phase contrast
FA: Focal adhesion
PCNA: Proliferating cell nuclear antigen
ERM: Ezrin, radixin and moesin
VASP: Vasodilator stimulated phosphoprotein
FBS: Fetal bovine serum
PBS: Phosphate-buered saline
TRITC: Tetramethylrhodamine
SEM: Scanning electron microscopy
TEM: Transmission electron microscopy
WT: Wild type
GEPIA: Gene expression profiling interactive analysis
EMT: Epithelial mesenchymal transformation
