# Supplementary material for: PTBP1 plays an important role in the development of gastric cancer
Source: Cancer Cell Int. 2023 Sep 5;23:195. doi: 10.1186/s12935-023-03043-0 (PMC10478210; doi:10.1186/s12935-023-03043-0)
Supplement: Supplementary file 1 — Genotyping Report [file 12935_2023_3043_MOESM1_ESM.pdf]

Supplementary File 1-Genotyping Report

集萃药康生物科技
GemPharmatech Co.,Ltd

Genotyping Report

Strain ID	T004617	Strain Type	KO(Cas9)	Genetic Background	C57BL/6J
Designer	Dongdong Zhang	Gene Name	*Ptbp1*		

1. Strategy of Genotyping

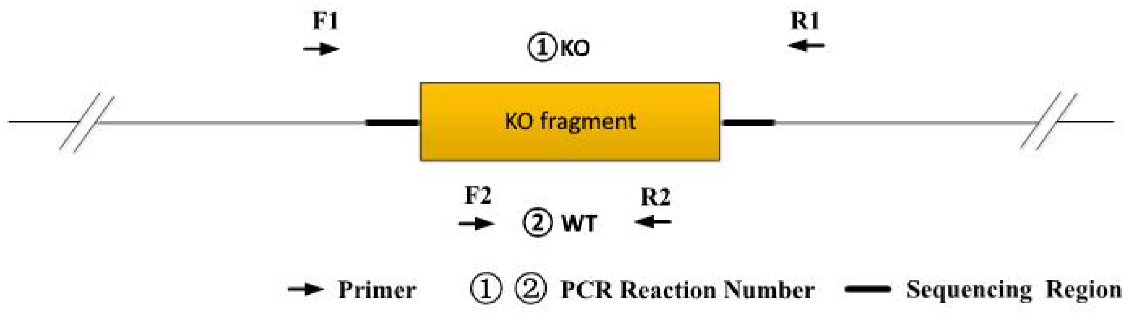

Wild type: ①PCR reaction obtains a single WT band; ②PCR reaction obtains a single WT band.
Heterozygote: ①PCR reaction obtains a WT band and a KO band; ②PCR reaction obtains a WT band.
Homozygote: ①PCR reaction obtains a single KO band; ② PCR reaction without product.
Note：1)The sizes of WT and Targeted band are shown below.
2)If the WT band is too large, it may not be possible to obtain a WT band.

2. Primer Information

PCR No.	Primer Name	Sequence	Band Size
PCR①	F1	TCAAGCTGAGTACCTATGGCAGTC	WT: 1595bp<br>Target: 780bp
	R1	CACTTATCTGAGGGAAGATCACCAG	
PCR②	F2	TCTGGTGATTCTGTCTTTGTGCTG	WT: 556bp
	R2	CACTTATCTGAGGGAAGATCACCAG	

3. Gel Image

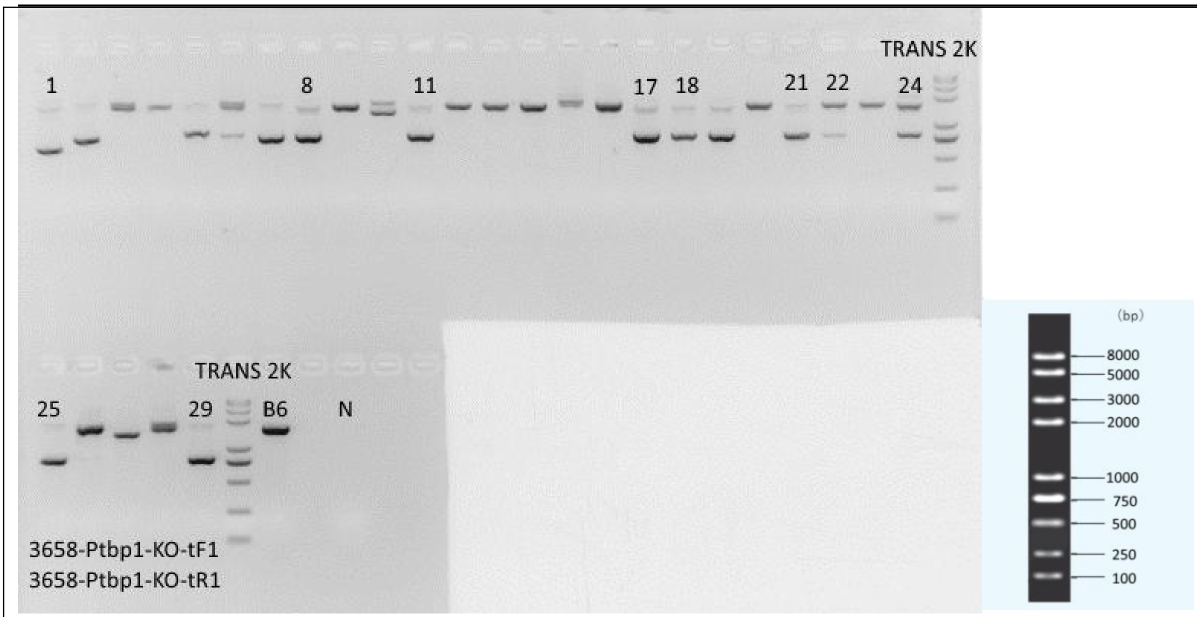

Note: B6: Wildtype control; N: Blank control (ddH_2O); TRANS 2K: DNA Ladder

4. PCR Condition

PCR Reaction Component		
**Seg.**	**reaction component**	**Volume (µl)**
1	2 × Taq Master Mix， Dye Plus，（Vazyme P112-03)	12.5
2	ddH2O	9.5
3	Primer A(10pmol/µl)	1
4	Primer B(10pmol/µl)	1
5	Template(≈100ng/µl)	1

PCR program

Seg.	Temp.	Time	Cycle
1	95℃	5min	
2	98℃	30s	20×
3	65℃（-0.5℃/cycle）	30s	
4	72℃	45s	
5	98℃	30s	20×
6	55℃	30s	
7	72℃	45s	
8	72℃	5min	
9	10℃	hold	

Reviewer:Ting Sun

Date:2019-8-7
