# Supplementary material for: PTBP1 plays an important role in the development of gastric cancer
Source: Cancer Cell Int. 2023 Sep 5;23:195. doi: 10.1186/s12935-023-03043-0 (PMC10478210; doi:10.1186/s12935-023-03043-0)
Supplement: Supplementary file 2 — Ptbp1 Cas9-KO Strategy [file 12935_2023_3043_MOESM2_ESM.pdf]

Supplementary File 2-Ptbp1 Cas9-KO Strategy

Ptbp1 Cas9-KO Strategy

Designer:Xiaojing Li

Project Overview

**Project Name**	***Ptbp1***
**Project type**	**Cas9-KO**
**Strain background**	**C57BL/6J**

Knockout strategy

This model will use CRISPR/Cas9 technology to edit the *Ptbp1* gene. The schematic diagram is as follows:

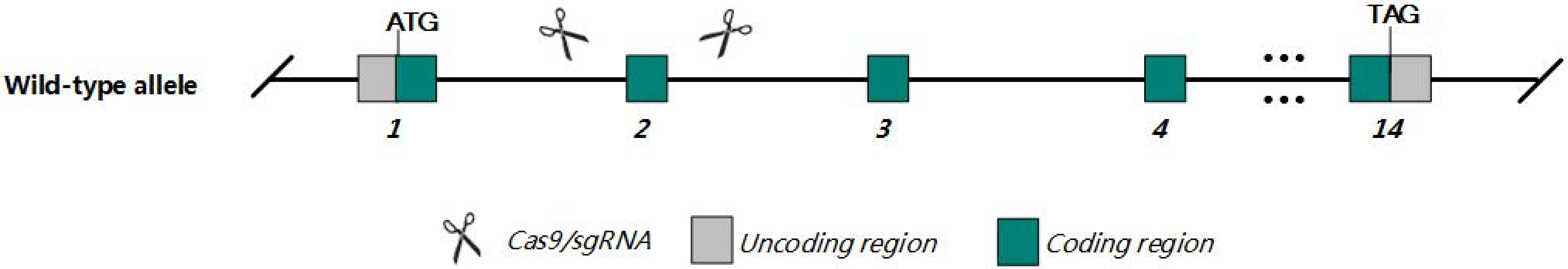

Technical routes

➢ The *Ptbp1* gene has 16 transcripts. According to the structure of *Ptbp1* gene, exon2 of *Ptbp1-216* (ENSMUST00000172282.7) transcript is recommended as the knockout region.The region contains 31bp coding sequence. Knock out the region will result in disruption of protein function.

➢ In this project we use CRISPR/Cas9 technology to modify *Ptbp1* gene. The brief process is as follows: sgRNA was transcribed in vitro.Cas9 and sgRNA were microinjected into the fertilized eggs of C57BL/6J mice.Fertilized eggs were transplanted to obtain positive F0 mice which were confirmed by PCR and sequencing. A stable F1 generation mouse model was obtained by mating positive F0 generation mice with C57BL/6J mice.

Notice

集萃药康 GemPharmatech

➢ According to the existing MGI data， Mice homozygous for a knock-out allele die before E6.5. Mice homozygous for a conditional allele activated in neuronal stem cells (NSCs) exhibit premature death, and non-obstructive hydrocephaly with loss of ependymal cells due to precocious NSC differentiation..

➢ The *Ptbp1* gene is located on the Chr10. If the knockout mice are crossed with other mice strains to obtain double gene positive homozygous mouse offspring, please avoid the two genes on the same chromosome.

➢ This Strategy is designed based on genetic information in existing databases. Due to the complexity of gene transcription and translation processes, all risks cannot be predicted under existing information.

Gene information （NCBI）

集萃药康 GemPharmatech

Ptbp1 polypyrimidine tract binding protein 1 [*Mus musculus* (house mouse)]

Gene ID: 19205, updated on 18-Jun-2019

Summary

**Official Symbol**	Ptbp1 provided by MGI
**Official Full Name**	polypyrimidine tract binding protein 1 provided by MGI
**Primary source**	MGI:MGI:97791
**See related**	Ensembl:ENSMUSG00000006498
**Gene type**	protein coding
**RefSeq status**	VALIDATED
**Organism**	Mus musculus
**Lineage**	Eukaryota; Metazoa; Chordata; Craniata; Vertebrata; Euteleostomi; Mammalia; Eutheria; Euarchontoglires; Glires; Rodentia; Myomorpha; Muroidea; Muridae; Murinae; Mus; Mus
**Also known as**	Ptb; PTB2; PTB3; PTB4; pPTB; HNRPI; PTB-1; AA407203; AL033359
**Expression**	Ubiquitous expression in thymus adult (RPKM 115.0), limb E14.5 (RPKM 113.3) and 28 other tissues See more
**Orthologs**	human all

Transcript information（Ensembl）

集萃药康
GemPharmatech

The gene has 16 transcripts,all transcripts are shown below:

Name	Transcript ID	bp	Protein	Biotype	CCDS	UniProt	Flags
Ptbp1-201	ENSMUST00000057343.15	1765	353aa	Nonsense mediated decay	-	E9QMW9	TSL:5
Ptbp1-202	ENSMUST00000095457.10	2598	489aa	Protein coding	CCDS70064	Q8CB58	TSL:1 GENCODE basic APPRIS ALT2
Ptbp1-203	ENSMUST00000099958.8	657	No protein	Processed transcript	-	-	TSL:3
Ptbp1-204	ENSMUST00000164385.7	693	No protein	Retained intron	-	-	TSL:2
Ptbp1-205	ENSMUST00000165153.7	2369	No protein	Retained intron	-	-	TSL:5
Ptbp1-206	ENSMUST00000165704.7	3169	529aa	Protein coding	CCDS35970	Q8BGJ5	TSL:1 GENCODE basic APPRIS ALT2
Ptbp1-207	ENSMUST00000165724.7	848	162aa	Nonsense mediated decay	-	F7AXP1	CDS 5' incomplete TSL:5
Ptbp1-208	ENSMUST00000168216.7	369	No protein	Processed transcript	-	-	TSL:3
Ptbp1-209	ENSMUST00000168683.1	375	55aa	Protein coding	-	E9Q0W3	CDS 3' incomplete TSL:2
Ptbp1-210	ENSMUST00000168988.7	950	No protein	Retained intron	-	-	TSL:3
Ptbp1-211	ENSMUST00000169091.1	319	107aa	Protein coding	-	F7DCW4	CDS 5' and 3' incomplete TSL:5
Ptbp1-212	ENSMUST00000169483.7	385	62aa	Nonsense mediated decay	-	E9Q279	TSL:3
Ptbp1-213	ENSMUST00000169580.1	2108	No protein	Retained intron	-	-	TSL:2
Ptbp1-214	ENSMUST00000171216.1	744	No protein	Retained intron	-	-	TSL:2
Ptbp1-215	ENSMUST00000171599.7	389	113aa	Protein coding	-	E9PZ39	CDS 3' incomplete TSL:2
Ptbp1-216	ENSMUST00000172282.7	3110	555aa	Protein coding	CCDS35969	Q922I7	TSL:1 GENCODE basic APPRIS P4

The strategy is based on the design of *Ptbp1-216* transcript,The transcription is shown below

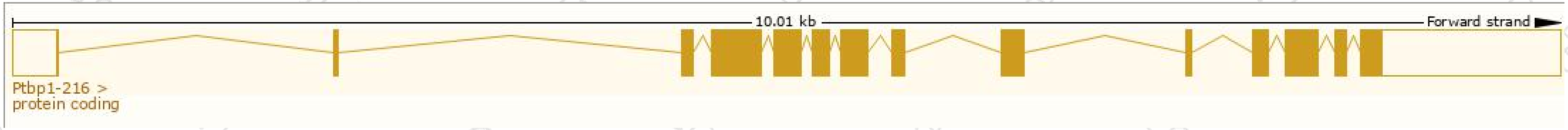

Genomic location distribution

集萃药康 GemPharmatech

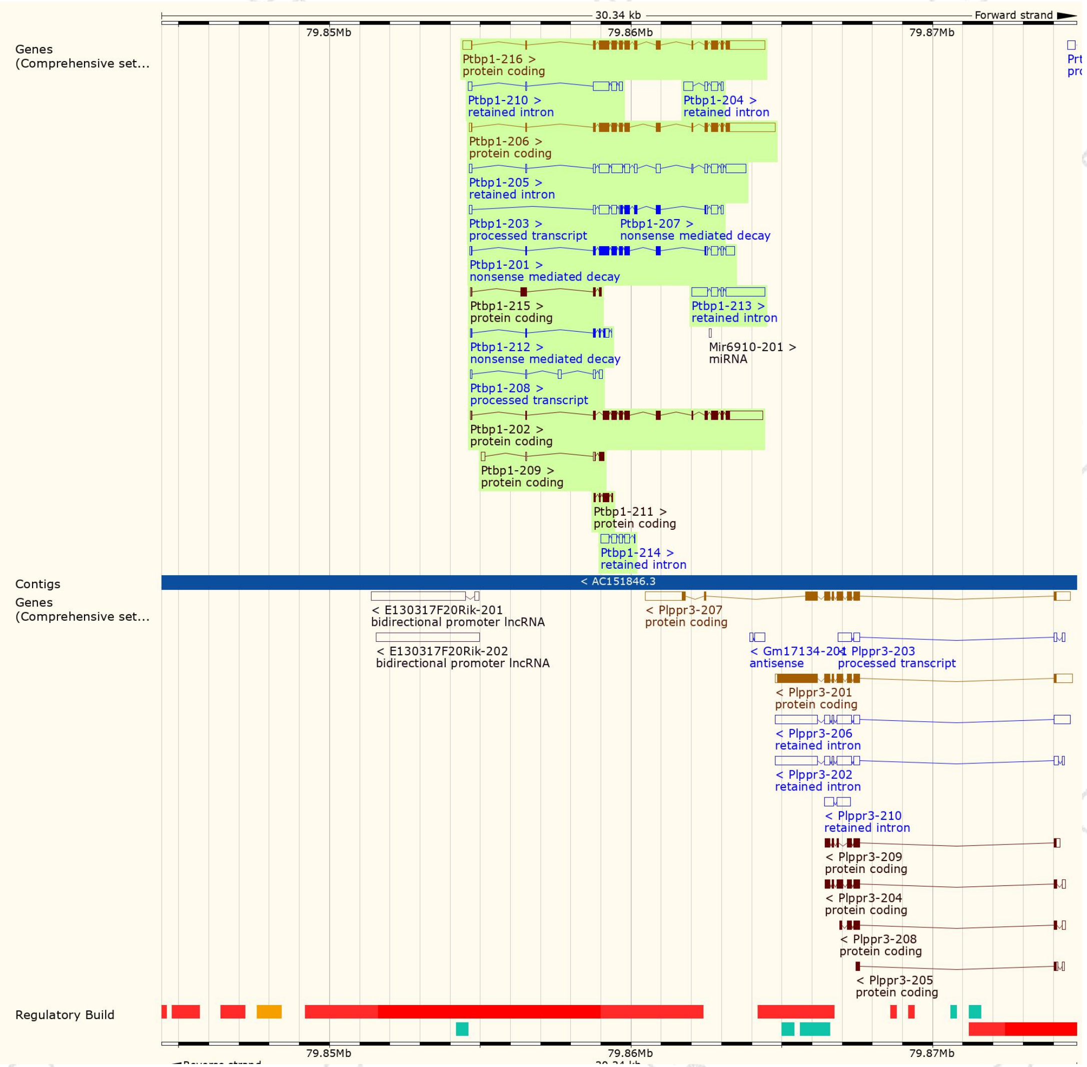

Protein domain

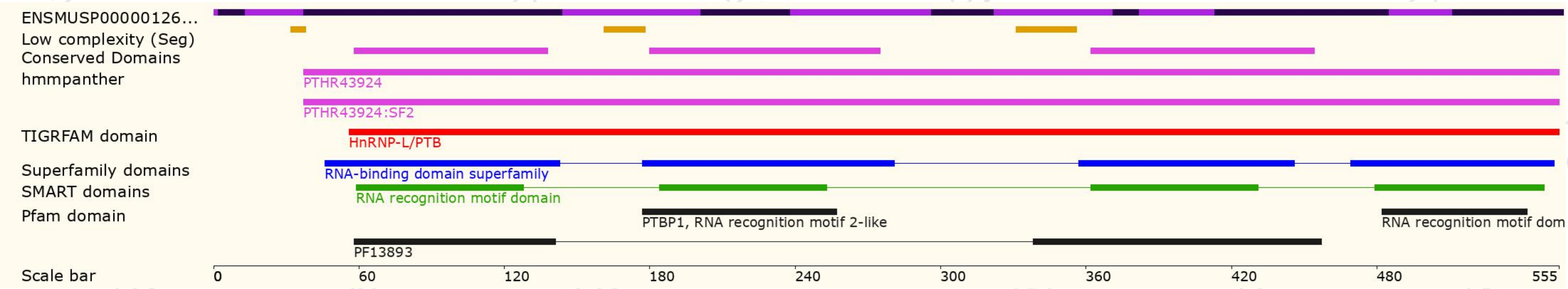

Mouse phenotype description(MGI)

集萃药康
GemPharmatech

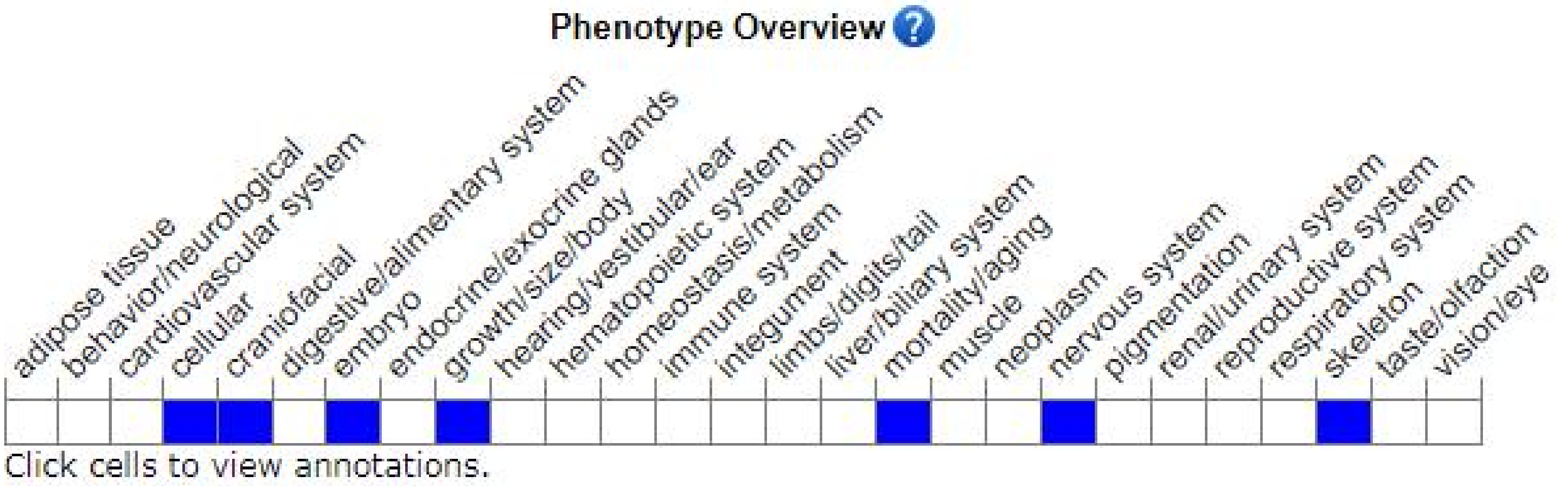

Phenotypes affected by the gene are marked in blue.Data quoted from MGI database(http://www.informatics.jax.org/).

Mice homozygous for a knock-out allele die before E6.5. Mice homozygous for a conditional allele activated in neuronal stem cells (NSCs) exhibit premature death, and non-obstructive hydrocephaly with loss of ependymal cells due to precocious NSC differentiation.

集萃药康
GemPharmatech

If you have any questions, you are welcome to inquire.

Tel: 025-5864 1534

集萃药康生物科技
GemPharmatech Co.,Ltd
