# Supplementary figures and images for: PTBP1 plays an important role in the development of gastric cancer
Source: Cancer Cell Int. 2023 Sep 5;23:195. doi: 10.1186/s12935-023-03043-0 (PMC10478210; doi:10.1186/s12935-023-03043-0)

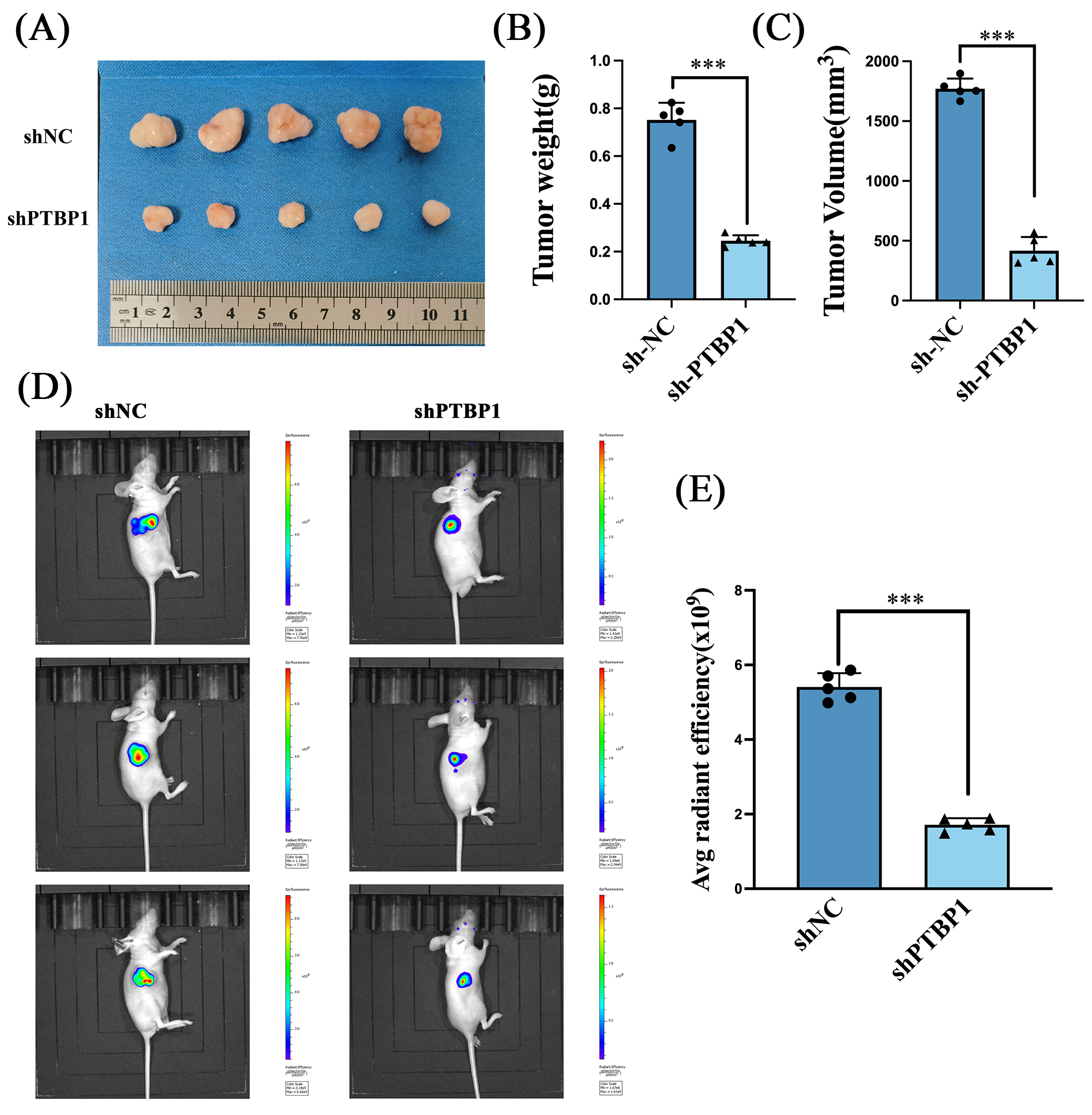

Supplement: Supplementary file 4 — Figure S1 [file 12935_2023_3043_MOESM4_ESM.png]
